# “ADAPTING TO A NEW REALITY”: OLDER ADULTS’ EXPERIENCES DURING THE COVID-19 PANDEMIC

**DOI:** 10.1093/geroni/igac059.1956

**Published:** 2022-12-20

**Authors:** Jodie Oshana, Mariana Guzzardo, Irina Todorova

**Affiliations:** University of Connecticut, Storrs, Connecticut, United States; California State University, Hayward, California, United States; Northeastern University, Boston, Massachusetts, United States

## Abstract

Older adults were uniquely affected during the COVID-19 pandemic, given that they were most at risk of serious complications and death if infected. Numerous preventative measures were abruptly mandated, including stay at home orders and social distancing that led to social isolation. Given this context, the aim of this qualitative study is to explore the lived experiences, perceived challenges, and mechanisms for resilience of older adults during the first wave of the pandemic. Our sample (n=50), derived from a larger international study with data from 15 countries, includes individuals 60 or older in Puerto Rico and mainland United States. Following thematic analysis of responses from an online survey, three themes were identified: resilience through reflection and adaptation, resilience through critique of systemic problems and injustices, and resilience through reaffirmation of values. Findings support various recommendations for improving support to older adults in future crises.

